# Understanding cancer patient cohorts in virtual reality environment for better clinical decisions: a usability study

**DOI:** 10.1186/s12911-023-02392-0

**Published:** 2023-12-20

**Authors:** Zhonglin Qu, Quang Vinh Nguyen, Chng Wei Lau, Andrew Johnston, Paul J. Kennedy, Simeon Simoff, Daniel Catchpoole

**Affiliations:** 1 School of Computer, Data and Mathematical Sciences, Western Sydney University, Sydney, Australia; 2 MARCS Institute and School of Computer, Data and Mathematical Sciences, Western Sydney University, Sydney, Australia; 3 School of Computer Science, University of Technology Sydney, Sydney, Australia; 4 Biospecimen Research Services, Children’s Cancer Research Unit, The Kids Research Institute, The Children’s Hospital at Westmead, Sydney, Australia

**Keywords:** Clinical Decision-making, Genomic Data Analysis, Usability Study, Virtual Reality, Visualisation

## Abstract

**Background:**

Visualising patient genomic data in a cohort with embedding data analytics models can provide relevant and sensible patient comparisons to assist a clinician with treatment decisions. As immersive technology is actively used around the medical world, there is a rising demand for an efficient environment that can effectively display genomic data visualisations on immersive devices such as a Virtual Reality (VR) environment. The VR technology will allow clinicians, biologists, and computer scientists to explore a cohort of individual patients within the 3D environment. However, demonstrating the feasibility of the VR prototype needs domain users’ feedback for future user-centred design and a better cognitive model of human–computer interactions. There is limited research work for collecting and integrating domain knowledge into the prototype design.

**Objective:**

A usability study for the VR prototype–-Virtual Reality to Observe Oncology data Models (VROOM) was implemented. VROOM was designed based on a preliminary study among medical users. The goals of this usability study included establishing a baseline of user experience, validating user performance measures, and identifying potential design improvements that are to be addressed to improve efficiency, functionality, and end-user satisfaction.

**Methods:**

The study was conducted with a group of domain users (10 males, 10 females) with portable VR devices and camera equipment. These domain users included medical users such as clinicians and genetic scientists and computing domain users such as bioinformatics and data analysts. Users were asked to complete routine tasks based on a clinical scenario. Sessions were recorded and analysed to identify potential areas for improvement to the data visual analytics projects in the VR environment. The one-hour usability study included learning VR interaction gestures, running visual analytics tool, and collecting before and after feedback. The feedback was analysed with different methods to measure effectiveness. The statistical method Mann–Whitney U test was used to analyse various task performances among the different participant groups, and multiple data visualisations were created to find insights from questionnaire answers.

**Results:**

The usability study investigated the feasibility of using VR for genomic data analysis in domain users’ daily work. From the feedback, 65% of the participants, especially clinicians (75% of them), indicated that the VR prototype is potentially helpful for domain users’ daily work but needed more flexibility, such as allowing them to define their features for machine learning part, adding new patient data, and importing their datasets in a better way. We calculated the engaged time for each task and compared them among different user groups. Computing domain users spent 50% more time exploring the algorithms and datasets than medical domain users. Additionally, the medical domain users engaged in the data visual analytics parts (approximately 20%) longer than the computing domain users.

**Supplementary Information:**

The online version contains supplementary material available at 10.1186/s12911-023-02392-0.

## Background

### Introduction

Human genome sequencing capacities continue to grow exponentially in volume, variety, and complexity [1]. Displaying a patient cohort so as to capture the complex genome and data analytics models reveals information about the individual. Genomic technologies have allowed rapid derivation of individual patient gene sequences and activity [2]. The increasing understanding of the complex human genomes leads to understanding individual rare and common diseases such as cancer. A clinical decision for the individual patient will be guided by understanding the biological features common to the disease type and unique to the particular patient [3].

Visual analytics is essential in genomics research as it can provide insight into biological processes, help users perceive correlations and trends in large datasets, and efficiently communicate findings to others [4, 5]. Machine learning and Virtual reality (VR) are both great technologies that present excellent opportunities; however, combining them will make various experiences more interactive and engaging for users [6, 7]. The field of healthcare discovery demands such visualisation tools not only for the precise and comprehensive representation of data but also for exploration leading to new insights and discoveries.

Capturing the complexity of genomic data to define the similarities and differences in a cohort of patients requires displays of high-dimensional models in low-dimensional (3D) space. VR is a technology that allows the user to explore and manipulate computer-generated three-dimensional (3D) environments in real-time to gain practical knowledge [8, 9]. VR has become more portable, immersive, and vivid, which has enabled the technology to be used in a broad range of medical applications [10], neurological disease, and other domains, including education [11] and construction safety [12]. The VR technology allows cancer specialists and analysts to move into the 3D space and explore the cohort for individual patients of interest. Viewing the spatial positioning of the individual patient in a 3D virtual genomic world enables clinicians to find patients’ genomic similarities and differences. The integrated machine learning algorithms would allow clinicians to uncover genomic relationships and inform decision-making for treatment regimens with more breadth and better accuracy [13, 14].

Improving the effectiveness of VR prototypes requires domain users’ assessment for further user-centred design and a better cognitive model of human–computer interactions. There is limited research work for collecting and integrating domain knowledge into the prototype design. Lau et al. developed a state-of-the-art work, called Virtual Reality to Observe Oncology data Models (VROOM) [15]. This prototype pioneers the integration of interactive visualisation, computational analytics, VR technology, visual design principles, game optimisation, and real clinical cancer data to assist decision-making in childhood cancer research. To support effective VROOM design, a pilot study with a small focus group of clinicians and biomedical researchers was carried out to collect domain users’ requirements before the development. VROOM allows domain users to identify each patient's unique genetic and biological traits and ultimately informs a clinician on deciding the best therapy for the individual.

Within our knowledge, there is unfortunately little usability study with the domain users on the effectiveness of VR works on observing oncology data models. This research is the first attempt to bridge the above gap by evaluating the feasibility of using VR for immersive data analytics of genomic data in domain users’ daily work.

Our study was carried out using VROOM where the domain users were asked to run the experiment using the VR device Oculus Quest™ to interrogate a patient cohort to assess the interrelationships between patient pairs and clusters. The participants were trained on how to use VR interactions, then they ran the VR application to manipulate the selection of patient information, comparison of patients exploring the similar or different data descriptors and gene expression values, with the comparison of selected patients from within the cohort. The feedback about the physical feeling, data dimensions in VR space, and usefulness in real domain work were collected. This article contributes:

i) A thoughtful usability study on the feasibility and effectiveness of VR for oncology data analysis with the domain users based on real clinical scenarios and tasks. The study addressed the potential design improvement in terms of efficiency, functionality, and end-user satisfaction. This pioneering work potentially establishes a guideline for future studies from quantitative results and qualitative domain users’ feedback and comments.
ii) Validate user performance measures derived from analysis of users’ feedback. We measured the task completion rate. The performance of different domain participants was also compared.

### VROOM prototype

**Fig. 1:**
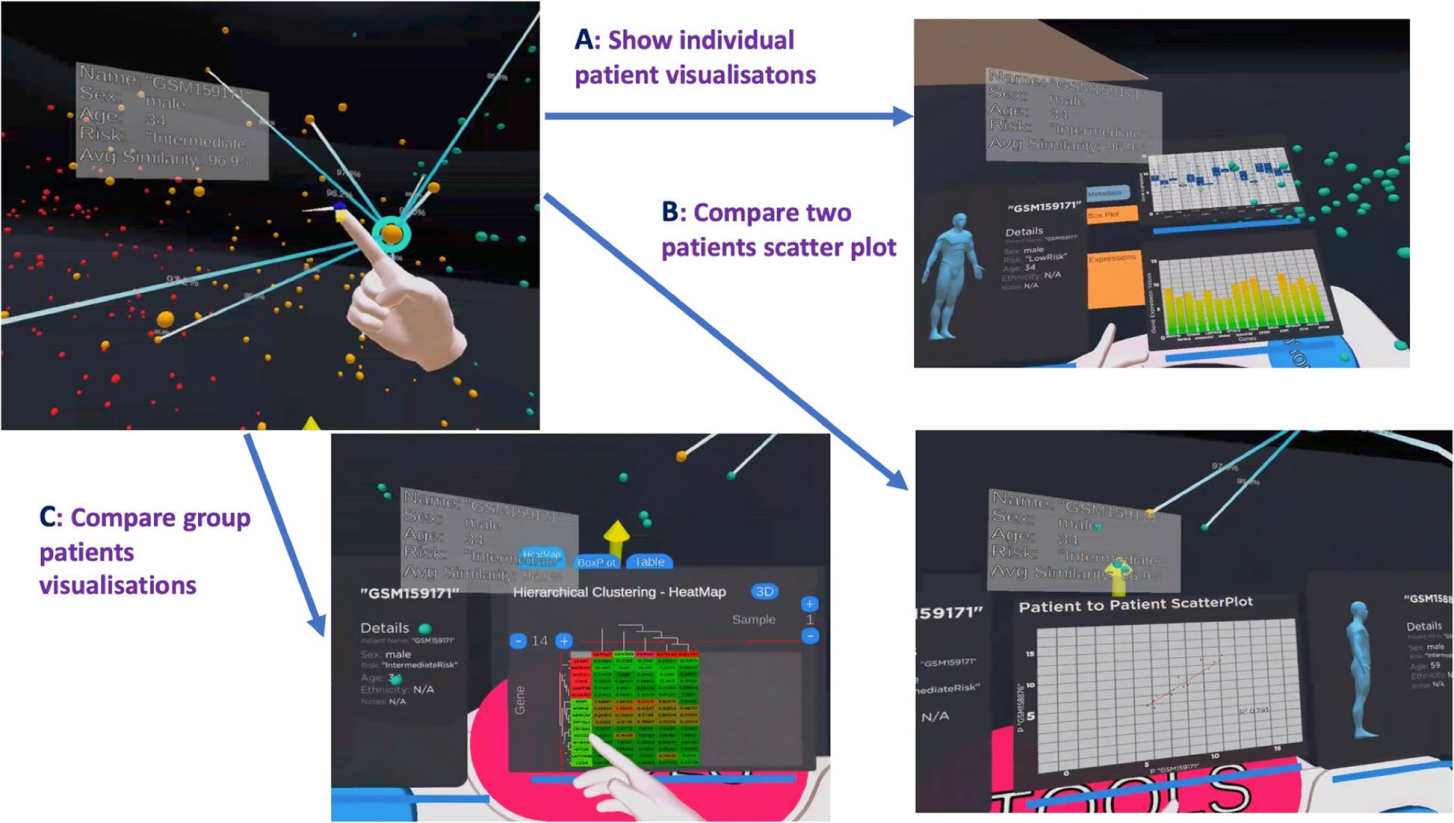
3D scatter plot for the entire patient population in the VR environment. The colour stands for the different levels of risk: red for high risk, green for low risk and orange for intermediate risk. This cohort uses “Hovon Three Risk” (http://www.hovon.nl/) data and the “Autoencoder” [16, 17] algorithm to decide the patient position. In this cohort, most high-risk (red) patients are clustered on the top right, some low-risk (green) and intermediate-risk (orange) patients are mixed in the middle, and a small group of low-risk (green) patients are also clustered together in the left bottom. Users can choose to **A**) show individual patient gene expression, **B**) compare two patients’ correlations, and C) compare multiple patients’ gene expression with heatmap [15]

With the requirement we collected from a preliminary study (present in Sect. "Preliminary study"), the Virtual Reality to Observe Oncology data Models (VROOM) [15] was developed for analysing cancer data. The VROOM system, as shown in Fig. 1, was used to demonstrate the analytical functionality for patient assessment. The primary purpose of this prototype was to help clinicians understand the Patient of Interest (POI) with the natural instinct to process and communicate data. With a VR environment, doctors could group similar patients based on genomic data and interact with the 3D visualisation. Each menu of VROOM opens a designed visualisation dashboard. When in “Individual Patient” mode, a single patient was selected, and a box plot and a histogram plot based on the patient’s genomic information were shown in Fig. 1A. When a clinician selected the "Patient to Patient" menu, a two-patient dashboard appeared in Fig. 1B. Finally, when selecting the "Patient to Group" menu, the third dashboard appeared as shown in Fig. 1C. These heatmaps, box plots, and dendrograms are domain users’ familiar visual charts and graphics. The relevant and sensible patient comparisons could assist a clinician with future clinical decisions. The sound was also designed and added to the visualisations to please and engage users.

The patient was visualised within the whole cohort in VROOM to map similarity and 3D location based on selected features and our designed machine-learning models. The location of the patient sphere was calculated with a machine learning dimensionality reduction (DR) [18] based on the selected genes by conducting an evidence-based feature selection. Different DR methods were used to transform, cluster and project patients into three-dimensional space for visualisation. The default algorithm was a network-based Autoencoder [16, 17], and the default dataset was “Hovon ThreeRisk” data [19] which is the Acute Myeloid Leukaemia data with the expression signature called leukaemia stem cell-17 (LSC17) analysis score [20]. All the data are in the public domain and in the form of bulk ribonucleic acid (RNA) sequencing data with patient attributes and history. Users could also choose one of the provided machine learning clustering algorithms such as the principal component analysis (PCA) [21], t distributed stochastic neighbour embedding (tSNE) [22], and uniform manifold approximation and projection (UMAP) [23]. They were also provided with the opportunity to choose a transcriptomic dataset from the data collection, including TCGA Research Network [24] and National Cancer Institute Office of Cancer Genomic—Target dataset [25] and a dataset consolidated and used by Tyner et al. [26] which is a whole-exome sequencing of 672 acute myeloid leukaemia samples (with 454 matched normals) from the Beat AML program.

The subsequent parts are organised as follows. Sect. "Method" presents the usability study method, including a preliminary study which was carried out to identify domain users’ requirements and lists the scenario, tasks, and procedures for the usability study’s experiments and participant recruitment. The analysis of the user feedback is presented in Sect. "Results analysis". We used various methods to analyse the feedback data and to find helpful information for future development. Sect. "Discussion" discusses the comfortable issues and the four areas: technical area, design method, visual analysis, and clinical utility. The final section, 5, closes with a conclusion and future work.

## Method

### Usability study

The usability study was implemented for the VROOM prototype with the users who would benefit from the use of the VR application. The users included hospital clinicians, genetics domain researchers and engineers, and some research students. The usability study was run in a lab at The Children’s Hospital at Westmead, which had enough space for portable VR devices, a laptop table, and video equipment to capture the user’s physical movement data. The participants were asked to complete routine tasks based on a real clinical scenario. Sessions were recorded and analysed to identify potential project improvement areas. The facilitator monitored the participant’s interactions with the VR application in the same office, wrote the notetaking, a data logger(s) on the system, and monitored videos in the same room. During the one-hour usability study time, four activities: signing the consent form, learning VR gestures, running VROOM, and giving feedback were run. The sixteen tasks, as shown in .

Table 1 were designed to cover all the prototype features and follow a clinician’ familiar scenario. The feedback from the 20 participants was analysed in Sect. "Results analysis".

**Table 1:** 16 tasks in this task list are run to solve a clinical scenario

Investigate process	Tasks
Navigate the scatter plot to feel comfortable	1. Zoom in/out the patient cohort
	2. Move the patient cohort
	3. Rotate the scatter plot
Choose your patient of interest (POI), e.g. a high-risk patient	4. Select a patient
Check the selected patient’s gene data visualisations. And compare with another patient’s information	5. Open an Individual Patient and see the patient details
	6. Change to another patient with similar gene data (a neighbour patient) or very different gene data (a faraway patient)
Compare the patient with another far away patient, and a neighbour patient	7. Open the Patient-to-Patient panel, drag two patients to patient comparison and compare two patients
	8. Change patient to compare different pair of patients
Mark a patient for future use	9. Mark a patient
Compare the marked patient with a similar group, check the heatmap, boxplot and table list. Choose a faraway group to compare. Find the marked patient to investigate	10. Open a patient to group comparation panel, compare one patient to a group of patients
	11. Show a heatmap, show 3D heatmap, modify the hierarchy
	12. Show the box plot, a table list plot
	13. Clear patient list and drag other patients
	14. Choose a marked patient
Choose another dataset and model	15. Choose another dataset and another machine learning model
More explorations	16. Use the new dataset and new model to repeat some of the above steps 1–13

### Preliminary study

Before the VROOM was designed and developed, a preliminary study was carried out to collect domain requirements. The broad research themes assessed how VR can make it possible to explore complex data models capturing genomic similarities and differences between childhood cancer patients.

We interviewed five domain experts and let them use three different genomic data visualisation tools on a personal computer, a mobile device, and a VR environment. Depending on their roles, the various users used the tools differently and gave some quotes and suggestions. One of them mentioned that genomic visualisation tools took out the complexity of the genomic data and simplified the patterns, which was very useful for the current work. The participants who worked for targeted medicine and personalised treatment research indicated that genomic and cancer data visualisation tools had the advantage of recognising patients’ data in a cohort and finding suitable treatment methods accordingly.

The pilot group also mentioned the other features they wanted to see in the genomic and cancer data visualisation tools. Firstly, they hoped to see more information on only one screen instead of jumping among different windows, which made presenting information among different roles easier. Secondly, users needed to add new data easily and see more detailed information in a few steps. Thirdly, the users indicated the need for Artificial Intelligence (AI) to predict features that could assist their decisions. Lastly, more health workers accepted applications run on VR devices, and more visualisation features were expected to be added to such applications. We incorporated all the suggested features into the VROOM, including design principals with sound in VR, providing additional visual charts to support the analytics, adding more computation analytics, and enhancing the interaction with more straightforward navigation.

### A clinical scenario and the task list

#### Scenario

When clinicians have a new patient, they want to compare the new patient’s genomic data with the existing patient population to understand the patient of interest (POI) in a more natural way. They need to find genomic similarities or different other patients, compare their genomic data, and understand the new patient similarity among the whole cohort. The purpose of this analysis is to identify the clinical needs of the new patient by comparing the patient's genomic data with other patients.

#### Task list

Based on the above scenario, 16 tasks are designed, as shown in Table 1 to cover all the prototype features and solve a real clinical question. The complexity of the tasks in the study reflected the potential users’ daily workflow.

These tasks were also ordered based on their working habits.

### Participants

Recruitment happened through direct email contact and word of mouth. The 20 participants were recruited, with 10 males and 10 females from different medical or computing backgrounds. The participants were potential end-users of the tool.

Due to the focus of our study on the domain users, the participants were hospital clinicians, genetics domain researchers and engineers, and some research students. The 20 participants mixed with young and old age; for example, clinicians were often old participants, and research students were young participants. The participants also have different VR experiences; for example, all clinician participants had no VR experience as they were usually not young and had no time to play modern computer games, while the group of invited research students had some or at least a little bit of VR experience as they had some chances to play VR games or other games that had similar controllers. We divided the participants into two main groups “Medical Domain Users” and “Computing Domain Users”. Clinicians and genetic scientists/researchers are grouped as “Medical Domain” users, and bioinformaticians and research students are grouped as “Computing Domain” users.

Before the study, the participants were asked to rank and describe whether and how much they were familiar with using Virtual Reality (VR) for playing VR games or had experience in using VR for data analysis. 60% of the participants had no VR experience, 15% of participants had a limited VR experience, 15% of participants had some VR experience, and only 10% of participants were highly familiar with VR. No one was a very professional VR user.

The diversity of participant backgrounds and VR experience levels allowed us to get less biased outcomes as well as analyse further the performance among different user cohorts.

The participants were then invited to attempt to complete a set of representative task scenarios presented to them in as efficient and timely a manner as possible and to provide feedback regarding the usability and acceptability of the user interface. The participants were directed to provide honest opinions regarding the usability of the application and to participate in post-session subjective questionnaires and debriefing (see more details in Supplementary 2).

### Procedure

**Fig. 2:**
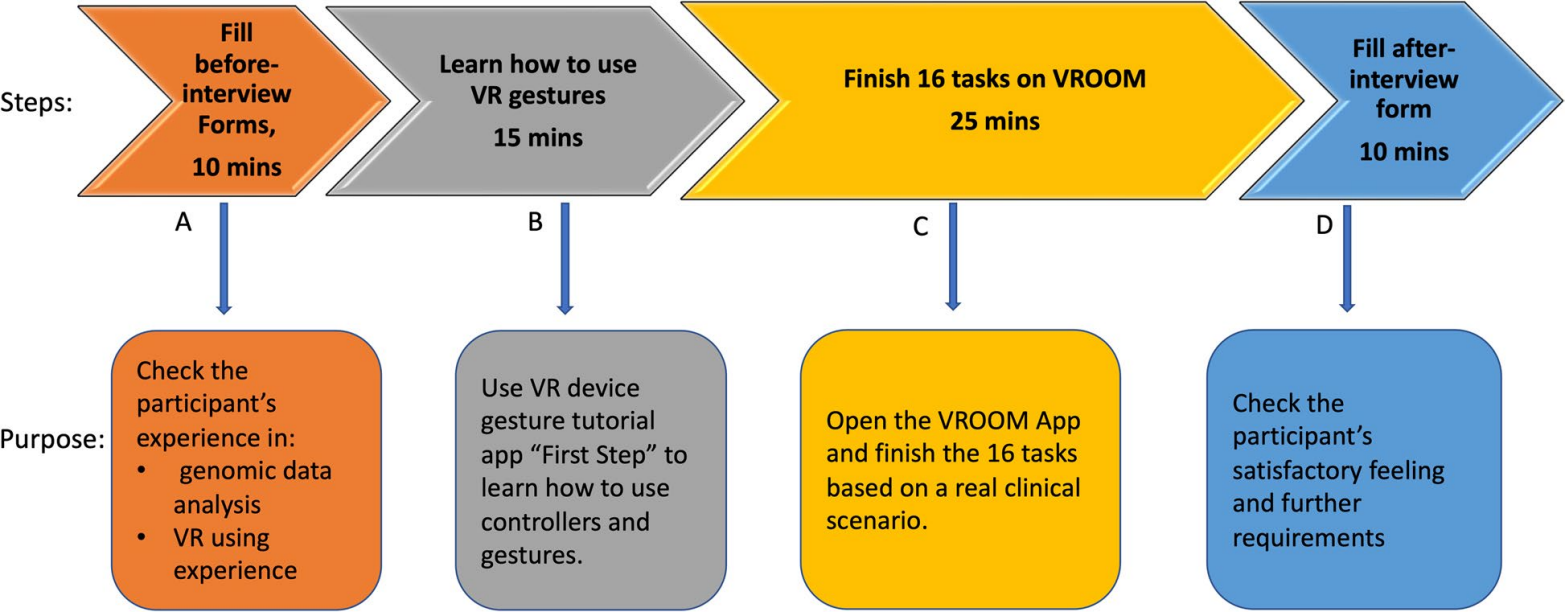
Usability Study Procedure: Activities and Expected Time. **A** Fill before-interview form, and consent form to check the participant’s experience in both genomic data analysis and using VR experience. **B** Learn how to use the VR device by using the VR device tutorial application “First Step”. **C** Finish a scenario with 16 tasks to find the scenario solution. **D** Fills the after-interview form for about 10 min to analyse users’ satisfactory feelings and collect users’ further potential requirements

We booked a two-hour slot with each participant just in case some participants needed more time. The study lasted for about one hour. During the one-hour study time, the participants and the facilitator followed COVID protocols for cleaning the device and sanitisation. Masks and social distancing were not required in our experiments. We do not consider them to be confounders in this study. As shown in Fig. 2, first, (Fig. 2A), the participants needed to fill out a before-interview form, and a consent form. This activity needed 10 min. The purpose was to check the participants’ experience in both genomic data analysis and using VR. Second, (Fig. 2B), the participants were given 15 min to learn how to use the VR device. We used an Oculus Quest gesture tutorial application to help users learn how to use controllers and gestures to control VR applications. The training session used some interactive games to teach the interactions. VROOM was designed with easy interactions and easy-to-perceive visualisations so that long training was not necessary for new VR users. Third, (Fig. 2C) the participants spent 25 min finishing a scenario with 16 tasks. This time could be extended if the participant wanted more time to plan the VR tool. In this step, participants were asked to finish all the tasks and find the scenario solution with the facilitator’s guide. Last, (Fig. 2D) the participants filled out the after-interview form for about 10 min. These questionnaire feedback were used to analyse users’ satisfactory feelings and collect users’ further potential requirements. Although the total study time was one hour, participants could spend a little bit more time if they liked to investigate more features by using a different dataset and algorithm.

### Analytical methods for results

We collected the user’s feedback and the time-to-completion of scenarios to measure the VROOM by using usability metrics. Usability metrics refer to user performance measured against specific goals necessary to satisfy usability requirements. There are many metrics to evaluate user experience. In this project, we used different methods to measure the effectiveness and satisfaction in a specified context of using VR for data visual analytics to achieve specified goals by specific users. The Mann–Whitney U test was used to analyse the tasks among different independent groups. Scenario completion success rates for effectiveness and task level satisfaction were used to communicate the results to stakeholders. The feedback texts are also analysed and visualised with a text mining program to find the most frequent words that participants mentioned and their relationships. Supplementary 1 provides the details on the analysis results and collected data.

Subjective evaluations regarding ease of use and satisfaction were collected via questionnaires, and during debriefing at the conclusion of the session. The questionnaires utilised free-form responses and rating scales. We asked the participants eight questions requiring rating scales and descriptions including usefulness, physical feelings, eye strain, arm fatigue, neck fatigue, comfort gesture, navigation in VR, clear text, avatar design, and sound. We analysed the results with rating scales visualisations, the trajectory of physical body movement, and feedback concept visualisations.

## Results analysis

### Scenario completion

Each scenario requested that the participant obtain specific data that would be used during a typical task. The scenario was completed when the participant indicated the scenario's goal had been obtained, whether successfully or unsuccessfully, or the participant requested and received sufficient guidance to warrant scoring the scenario as a critical error. With the facilitator’s guidance, no error rates were recorded in our study. We calculated the percentage completion rate as effective using the following formula.

$$\mathrm{Effectiveness}=\frac{\mathrm{Number \,of \,tasks \,completed \,successfully}}{\mathrm{Total \,number \,of \,tasks \,undertaken}}*100\mathrm{\%}$$

The completion rate in this usability study was 95% indicating most participants could successfully finish all the tasks during the study time. However, some spent more time on some tasks than others for a lack of VR experience.

### Scenario completion time and time-based efficiency

The time to complete a scenario is referred to as "time on task". This time (minutes) is calculated based on the video that participants saw in the headset. The time for each task started from the end of the last task and the start of the next task, and all the time was rounded to 0.5 min.

**Fig. 3:**
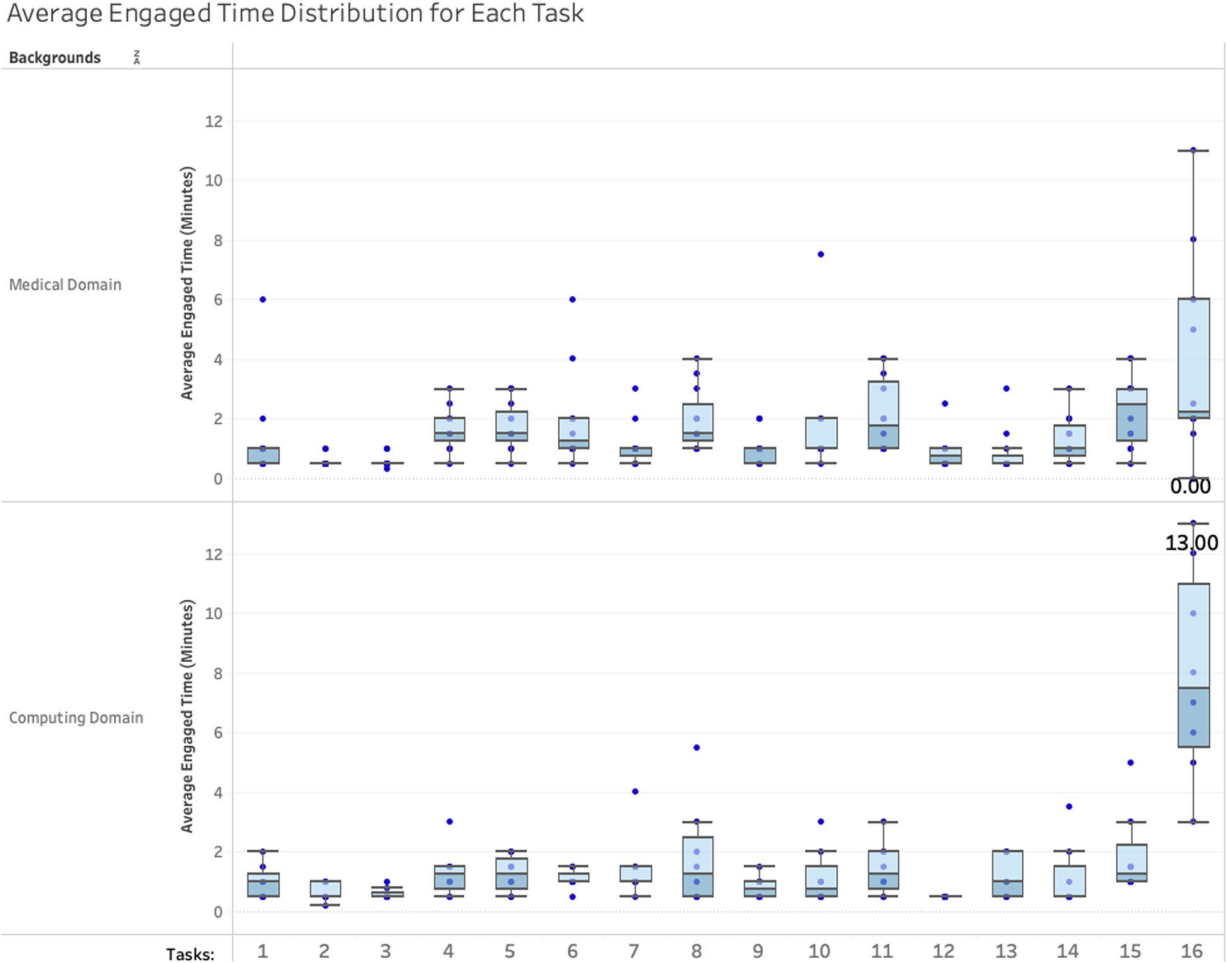
Time distribution for 16 tasks among two main groups: Medical Domain and Computing Domain

The time distribution for each task was also plotted in box plots shown in Fig. 3, indicating that each task had a different time distribution among the two main groups. For example, medical domain participants used a long time on task 11 but less time on task 16 than the computing domain users. For task 16, users took more time than the other tasks as it asked participants to repeat all the operations they had done with another dataset. Most participants were very interested in the VR tool features and spent lots of time repeating the whole scenario with another dataset or another algorithm for more explorations. The maximum time of this exploration task was 13 min, the average time was 5.6 min, and four participants spent more than 10 min repeating the features. These four participants were clinicians and genetic scientists, and they were very interested in the tool and could finish the other features in a shorter time and spent more time on the repeating task to further understand the data.

**Fig. 4:**
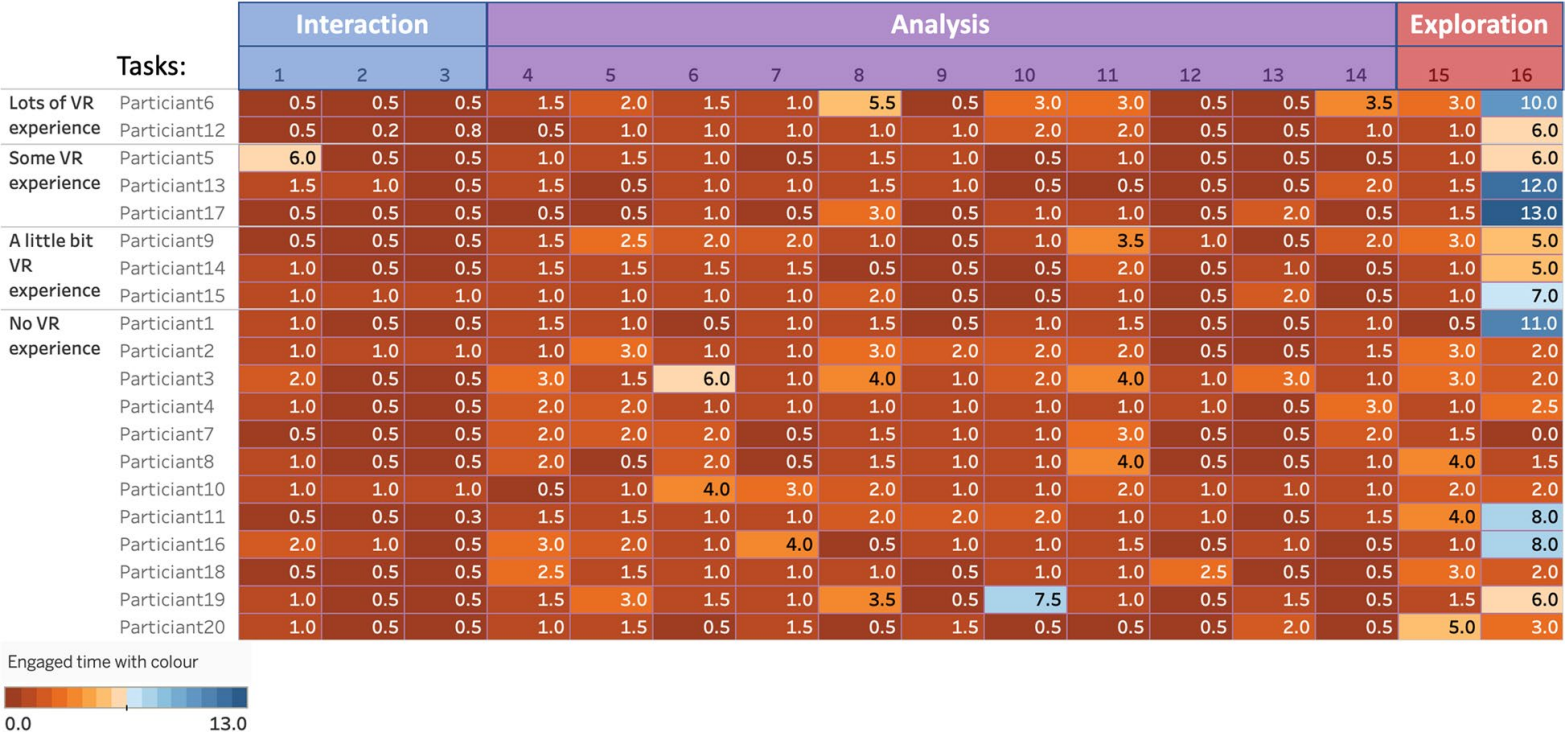
Engaged time of different VR-experienced participants. Tasks are grouped to three main tasks based on the purpose: Tasks 1–3 are “Overview Interactions”, Tasks 4–14 are the “Analysis”, and Tasks 15–16 are “Exploration”. The colour of the table cells stands for the average engaged time

As shown in Fig. 4, the first three tasks were to overview general navigation within the VR environment, including zooming, moving, and rotating the whole cohort. Participants usually spent less than one minute finishing the three tasks. There is only one participant who spent 6 min on task 1 to zoom in and out (see Table 1) as the participant pressed the wrong controller button and had to reopen the tool. The participants spent more time on the two main analysis tasks 8 and 10, as these two visualisations were what they were interested in more. Task 8 was for comparing two patients’ genomic expressions and task 10 was for comparing one patient with a group of patients. These two tasks were the main analysis in clinicians’ daily work. The figures in the dark blue cells of the last column indicate that if participants had VR-usage experience, they usually spent less time on the first several tasks but spent more time on the last task to explore more features with a new dataset as they usually learnt the features quickly to inspire them more interested in the tool.

We combined tasks together to create three main big tasks based on the purpose of the tasks: tasks 1–3 as “Overview Interactions”, tasks 4–14 as the “Analysis”, and tasks 15–16 as “Exploration”. The Mann–Whitney U test was used to compare the difference between two independent groups when the dependent variable was either ordinal or continuous, but not normally distributed.

We compared the average time the two main participant groups used for the three main tasks as shown in Table 2. To further compare the difference between the three main tasks among the two groups, we then used Mann–Whitney Test method to compare the difference and get results shown in Table 3:

i) Medical domain users running the Exploration tasks were statistically significantly different to the computing domain group (U = 16.5, *p* = 0.015). U reflects the difference between the two rank totals. Computing domain users spent more time (Mean = 9.875 min, SD = 3.2814) exploring the VROOM through changing algorithms and datasets than the medical domain users (Mean = 6.292 min, SD = 3.1076). This indicated that computing domain users might be more interested in VR feature exploration.
ii) The Medical participants engaged more time on data analysis tasks indicating they were more willing to try VR data analysis features to apply its use to their daily analysis and decision process. The different was not very significant (U = 28.500, *p* = 0.131).
iii) The time spent for the overall interaction had no big difference (U = 46.50, *p* = 0.905) between the two groups.

**Table 2:** Average time spent on the three main tasks between two different groups. The time highlighted with blue colour is more than another group

Compare Mean and Std. Deviation				
Two backgrounds		Overview Interactions	Analysis	Exploration
Medical Domain Users	Mean	**2.483 min**	**16.250 min**	6.292 min
	Std. Deviation	1.5503	4.8734	3.1076
Computing Domain Users	Mean	2.250 min	13.063 min	**9.875 min**
	Std. Deviation	.8018	4.2125	3.2814

**Table 3:** Mann-Whitney test statistics

Test Statistics^a^			
	**Overview Interactions**	**Analysis**	**Exploration**
**Mann–Whitney U**	**46.500**	**28.500**	**16.500**
**Wilcoxon W**	**124.500**	**64.500**	**94.500**
**Z**	**-.120**	**-1.511**	**-2.439**
**Asymp. Sig. (2-tailed)**	**.905**	**.131**	**.015**
Exact Sig. [2*(1-tailed Sig.)]	**.910b**	**.135**^**b**^	**.012**^**b**^
**Exact Sig. (2-tailed)**	**.939**	**.138**	**.013**
**Exact Sig. (1-tailed)**	**.476**	**.069**	**.006**
**Point Probability**	**.026**	**.006**	**.001**

^a^Grouping Variable: Two background

^b^Not corrected for ties

These results indicated that medical domain users interacted with VR tools use similar levels of ease to computing domain users. However, the medical domain users spent more time on the data visual analytics tasks than the computing domain users as these analysis tasks are the clinician’s daily work. The further exploration of the VR environment engaged computing background users longer as these tasks focus more on the algorithms and raw data.

### Subjective evaluation results

We asked the participants to rate and brief their feelings on eye, body, graph design, text, navigation, and sound. The questions were designed to gain further understanding of user experience, user preference, and additional comments for potential future design improvements.

**Fig. 5:**
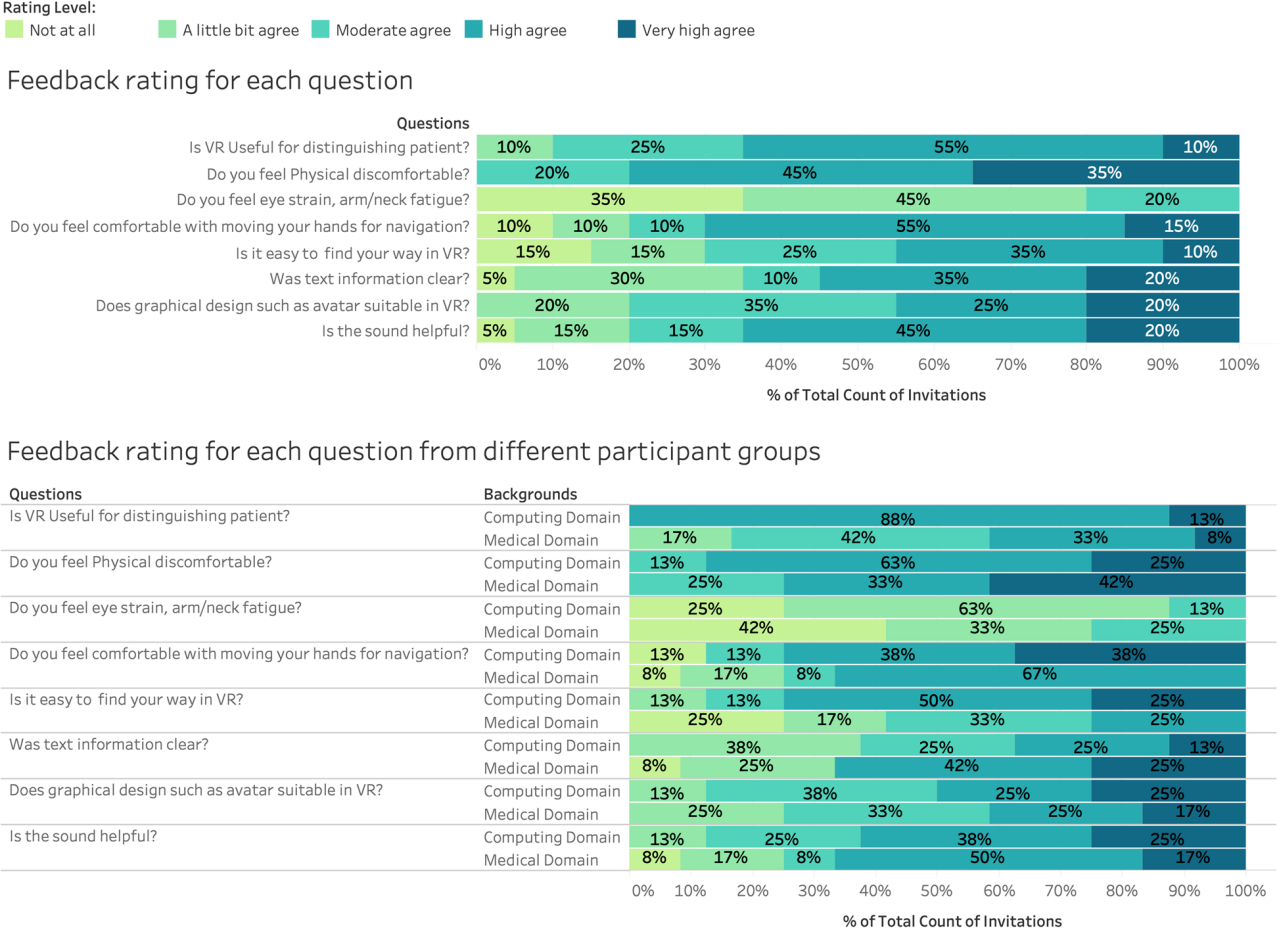
Feedback rating for each question in the whole group (top chart) and comparison between two main groups (bottom chart). The charts show the percentage of the rating in the whole group and between two groups

The overall rating scales were shown in Fig. 5 top chart, and the two background rating scales are in Fig. 5 bottom chart. We could see most participants rated very high or high degree for these questions. 65% of participants with high or very high agreed that the VROOM was useful for distinguishing patients, and this percentage was 41% among medical domain users. 80% of participants did not feel or only felt a little bit of eye strain, and arm/neck fatigue, and this number was 75% among medical domain users.

It was surprising that medical domain participants were more comfortable with body gestures, navigation in VR, and clear text in VR. 70% of participants with high or very high agreed to feel comfortable when moving hands for navigating in the VR environment, and medical domain participants had a higher rate of 75% on this scale. 45% of participants agreed that it was easy to find their way in the VROOM, while this number is 58% among medical domain users.

**Fig. 6:**
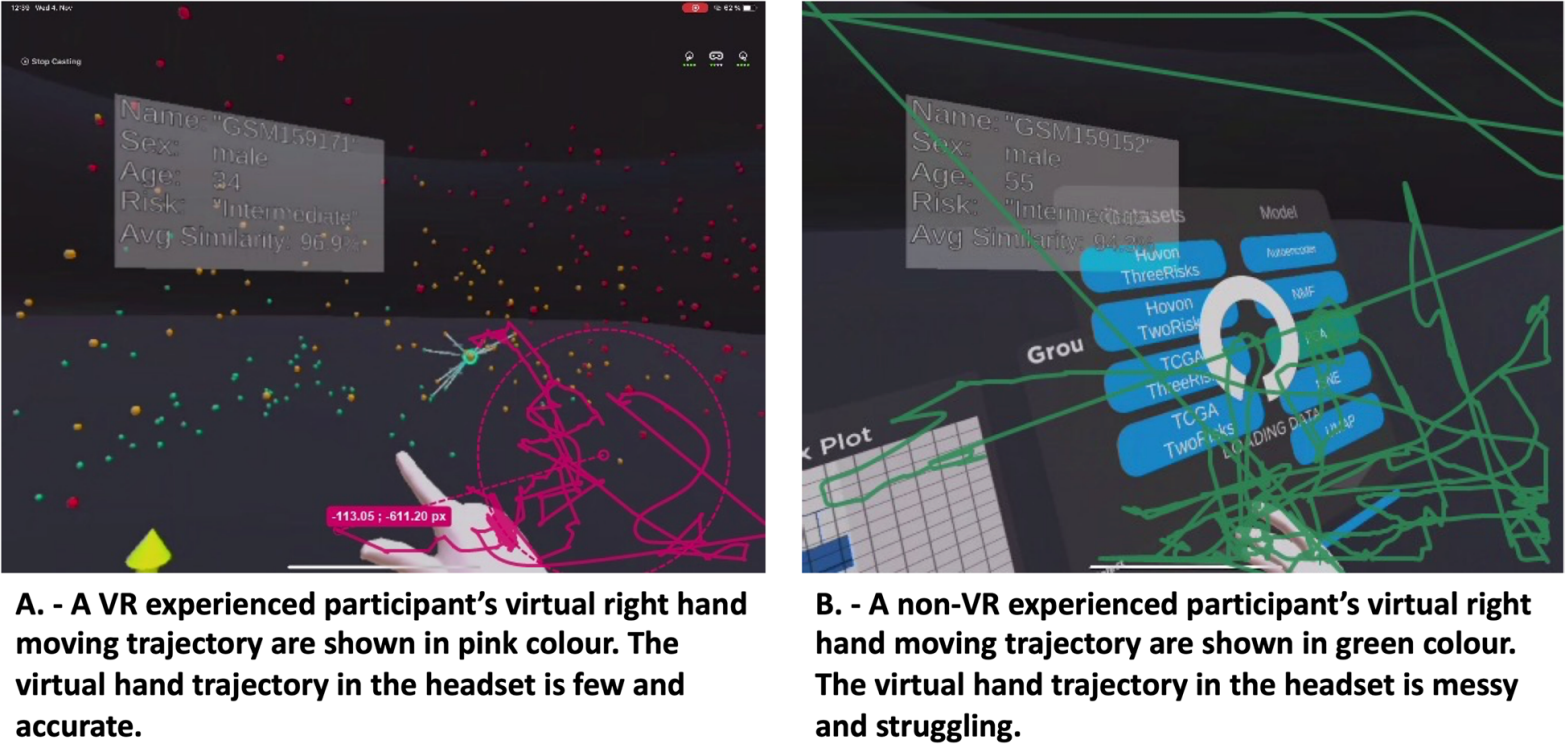
Trajectory for the virtual environment for a VR-experienced participant and a non-VR-experienced participant. The movement scope is in the human reaching scope. The participant can reach and interact with all the virtual objects in the movement scope without standing up

We also analysed the trajectory of physical body movement with two of the participants’ using computer assessment of captured videos: head, left hand, and right hand and virtual body: right hand. We used the tool Kinovea [27], which is designed for sport analysis, to analysis the movements. We used the motion trajectory feature to analyse two participants’ physical and virtual movements. One participant had plenty of VR experience and another one had no VR experience. The experienced participant’s right-hand movements were shown in pink colour as shown in Fig. 6A. The virtual hand trajectory in the headset is few and accurate. Figure 6B showed a non-VR experience participant trajectory. The virtual hand trajectory in the headset is highly varied indicate the user strugglingly to reach the targeted places. For both trajectories and observations, we could see the movement scope was in the human reaching scope. It was a controlled gesturing with all the movement limited to arm’s length stretches in front of him at desk height. The participants could reach and interact with all the virtual objects in the movement scope without standing up. There was no need for contortionist moves or massive body extensions. It demonstrated comfort as the participants managed the space around them and in front of them.

For the clear text information, 55% of participants had high or very high agree rates, and medical domain users were 67%. The participants rated how good the graphical design including the avatar design. 45% of the participants rated it as high or very high, this number is 42% among medical domain users.

The prototype added designed sound to the data visualisations with the aim to assist the user interactions in the immersive environment and create a sense of connection with the visual cues [28, 29]. VROOM had three main sonic components: data sonification, user interaction with visual elements and ambient background sound. Five guiding principles for each sonic component were assessed: aesthetics, congruence with action, congruence with visual elements, having a distinctive signature and are spatially informative. The purpose of the sound was to aid the users in navigating and interrogating data points in an immersive environment which was pleasant and harmonious. 65% of all participants with high or very high agreed that sound was very helpful while this number was 67% among medical domain users.

### Feedback concept analysis

**Fig. 7:**
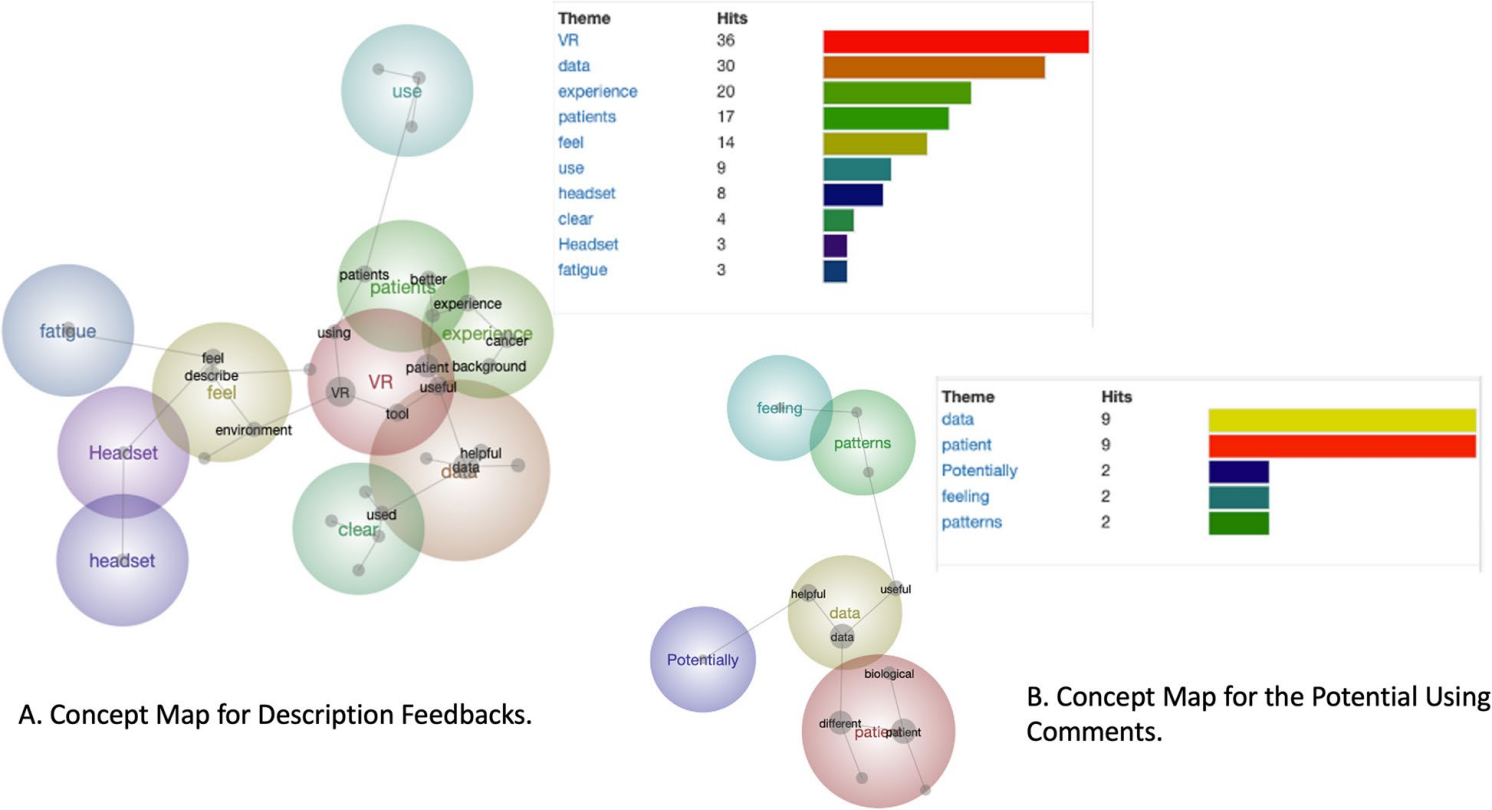
Concept map for description feedbacks (**A**), and the potential using comments (**B**) to show a list of concepts contained in the text, and their relationship to each other

All the participants’ description feedback was collected and analysed with the text-mining approach. Some limitations and challenges might be associated with subjectivity in interpreting free-form responses. We used the text mining program Leximancer [30]. The tool provides concept maps which contained the main concepts that occur within the text and their relationships. Figure 7A shows the concept map for the relationship between the comments. Most mentioned words are “VR”, “data” and “experience”. The Fig. 7B shows the concept map for the future suggestions. Most mentioned words are “patient”, “pattern” and “potentially”.

Most words were positive such as “useful” and “clear”. Participants spoke multiple times about “headset” as it was a little bit heavy for some participants. The negative word “fatigue” was also mentioned several times by users. We then checked the detailed comments and find the VR prototype's strengths and weakness from the participants’ feedback comments.

When asked about the usefulness of the VROOM, the participants mentioned the strengths of the VROOM are: “A better experience, relevance to daily work, useful for 3D visualisation, easy to see, clearer to clinicians, a better view on the data, many dimensions and vectors,” with some need-improvement suggestions: “Needs to be able to load other data, need time to learn, need more explanation”.

The physical discomfort question received some positive feedback, such as “no motion sickness, didn't feel sick” and negative feedback such as “Heavy headset, discomfort on the eyes, need time to familiarise with the environment, slightly discomfort”. Participants felt differences on the eye strain and arm/neck fatigue, but most of them felt better after practice and getting used to the VR environment. The participants gave feedback on the visualisations, navigation and interactions in the VR environment.

They gave additional comments about the strengths for using VR, such as “Definite learning curve, Easy, become easier with practice, simple; good and appear relevant, clear; Good avatar, Good panel design, quickly and easily show gender, help to recognise patients”. Some negative feedback was also given, such as “Hard to pick dots, practice and adaptation required; Some small texts, a little bit blur with glasses on, a little blurry, justify headset to make clear; Too big console, not friendly colour blindness, use of cube to mark patients may not be useful, being able to add additional info to avatars potentially useful”. The designed sound only got some positive feedback:” Helpful, nice sound, engaged background music, help to confirm the action, adds to the VR experience” without any negative comments.

Participants also provided detailed comments on how the VR tool might be used to help researchers or medical doctors make more sense of the complex data comparisons when trying to understand a particular patient. We also checked the detailed comments and find the VR potentially use in medical domain users’ daily work. The participants mentioned the strengths of the VROOM were: “Helpful, improve complex data, feeling emerged/pattern in the data, potential to be used in a practical setting, useful to identify interested patient, useful for data comparison, useful for data comparison, intuitive understanding, intuitive understanding, useful in multiple fields, useful in multiple fields, useful in multiple fields, great benefit, complex 3D relationship” with some need-improvement suggestions: “Using different parameter – Clinical ones (e.g. response to therapy, important to have clear clinical questions to answer”. The users gave some detailed improvement comments, such as “adding more information to avatars”, “simplifying the user interface”, and changing the used way of the controller buttons.

## Discussion

### Comfortable use and safety with cybersickness issues

Our study stressed further the maximum continuous usage time on VR of around 30 min before causing to loss of spatial awareness of the room around the users [31]. VR Space [32] suggested taking a break from the VR environment to recover and re-adapt to the real world. The VR game players are suggested to take a 15-min break for every 30 min of play. In this usability study, we let participants continuously use VR for about 40 min, including training time and using the VROOM tool time. After they took off the headset, they estimated the maximum time they could use VR. Surprisingly, most clinicians thought the average of 45 min was the time they could endure the VR environment, while the other three groups, computer, bioinformatics, engineers, genetic scientists/researchers, and students thought an average of one hour was OK for them. The estimated time (45 min or 60 min) was longer than the recommended time (30 min) in [31]. The possible reason was that this tool was for data analysis to make users focus on solving problems instead of repeating game interactions. The participants might forget the different VR spaces when they concentrate on problem-solving.

Existing research is on assessing eye fatigue caused by the VR environment from different angles, such as head-mounted displays using eye-tracking [33] and the Ocular effects of VR headsets on young adults [34]. Oculus handbook stated that one in 4,000 might suffer dizziness, seizures, and eye or muscle twitching and recommended that users suffering these symptoms discontinue using the headset and see a doctor [35]. In our usability study, all 20 participants finished the whole study without indicating an experience of eye strain. Some of them felt slightly uncomfortable with eyes out of focus, mild neck fatigue, and dizziness suggesting the VROOM prototype can be operated within safe user requirements.

### VR in the technical area, design method, visual analysis, and clinical utility

VR for data analysis differed from the game industry to allow users to have fun. VR for data analysis should enable users to explore the information quickly and effectively. In this usability test study, all the participants were potential users, such as clinicians, biological background researchers, childhood cancer researchers, and genomic data researchers. They provided precious feedback and recommendations. We analysed the feedback in four areas: technical, method of design, visual analysis, and clinical utility to find what domain users wanted for VR use in the data analytics area in the medical field.

For the technical area, we studied how VR was used for genomic data analysis for Acute Myeloid Leukaemia cancer. The participants were interested in the VR space and were familiar with the clinical scenario that the prototype presented. As one of the participants mentioned, the VR natural interactions and the sounds could engage cancer clinicians in a new way. The participants were curious about what more VR data visualisation could do. Some commented that they wanted to use their research or clinical data to try this exciting platform. Participants had a high completion rate of 95%, and most of them could finish all the designed tasks in time. We also calculated the time accuracy for each task among different participant groups. We grouped them into medical and computing domain users and the tasks into interactions, analysis, and explorations. All participants could quickly learn how to interact and engage in a VR environment. The medical domain users spent more time on data analysis tasks, while the computing domain users spent more time on functional features.

For the method of the design area, the user experienced and judged the user interface design and gave feedback. The participants felt strange and excited when using VR 3D space for the data analysis. They also felt surprised when they found that they could visualise the graphical representation of complex data from an immersive point of view, press a button, and select an icon like in the real world. They commented on the colour, the avatar design, and the sound based on their personal experience. The most comments were on VR space design, especially how the cohort was presented and interacted with and the console location. We plotted the trajectory of physical body movement with two of the participants videos. The movement scope was in the human reaching scope. The participant could reach and interact with all the virtual objects in the movement scope without standing up. The colour could also be improved to get colour-blind friendly because one participant is red-green colour blind and could not recognise the patient based on the colour.

For the visual analysis area, the integration of different data analysis strategies was investigated. The prototype combined data visualisation, machine learning and game optimisation to make the visual analysis results useful. The participants valued the visual analysis methods, especially the 3D scatter plot for cohort display, the linear correlation for the two patients’ data comparison, and the heatmap for group comparison. All the genomic data visual analytics methods were designed based on a preliminary study with the end-users to ensure they met the actual requirements and needs. The participants asked many questions about how to get the visualisation results, including algorithms and features. More annotations should be added to the visual analytics results.

The last area was the clinical utility which investigated the feasibility of using VR for genomic data analysis in domain users’ daily work. The usability scenario was very close to the actual procedure in cancer clinicians’ daily work. What the clinician need was straightforward; for example, they compared the new patient genomic data with some existing data and understand the new patient’s clinical need. The traditional way was to give the data and tell the needs to a data analyst or did it by themselves and then got the related analysed results to assist their decision-making process. The VR prototype potentially improved their work by providing a more engaged environment and getting the results quickly and simultaneously. Still, it needed more flexibility, such as allowing them to define their features for the machine learning part, adding new patient data, and importing their datasets as one participant mentioned. VR devices such as headsets and controllers still needed to get more convenient such as lighter and fewer buttons, to let users quickly and comfortably get helpful information.

## Conclusion, and future work

The usability study investigated the potential of using VR for genomic data analysis in domain users’ daily work. The study demonstrated that VR technology, specifically VROOM, facilitates the visual data analytics of genomic data across patient cohorts and the feasibility for decision-makers such as clinicians and researchers to assess individual patients within the cohort. We employed various analytical analysis and measurement methods to find insights into the results.

We measured effectiveness in a specified context of using the VR prototype to achieve specified goals by specific users. Scenario completion success rates for effectiveness and task level satisfaction were used to communicate the results to stakeholders. The Mann–Whitney U test was used to compare the difference between medical domain participants and computing domain participants. Both the medical domain and computing domain users could quickly learn how to use VR but had a different focus when trying the VR prototype. We also discussed issues on comfortable use and safety with cybersickness issues. VR for data analysis in four areas, technique, design, visual analysis, and clinical utility, were also discussed.

The future work could be on ergonomic assessment in a virtual environment to make the VR space design more data-driven and user-centred. Moreover, the genomic data visualisation user interface also needed to improve to better use the VR 3D more natural way of interacting. Lastly, the genomic data analysis needed to combine different analysis strategies, such as machine learning and add suitable explanations to make the results more explainable, trustable, and interpretable for domain users. More human-centred studies will be carried together with new and more capable extended devices from different providers as such the devices are getting lighter and the technologies are getting more user-friendly. Current participants were recruited through direct email contact and word of mouth from a hospital and two universities. This bias might impact the generalisability of the study results. Large-scale investigations with a larger number of participants and analytical and communication features will also be carried out in the future. Prolonged study, especially in a clinical setting, will be carried out in the future.

### Supplementary Information

**Additional file 1: Supplementary 1. **Supplementary tables for the analysis of usability results.

**Additional file 2: Supplementary 2. **Questionnaire.

## Data Availability

The data can be provided subjection to the ethics approval’s restriction. The main contact persons are Z.Q. and D.C.

## Abbreviations

VR: Virtual Reality
VROOM: Virtual Reality to Observe Oncology Data Models
SD: Standard Deviation
PCA: Principal Component Analysis
tSNE: T Distributed Stochastic Neighbour Embedding
AI: Artificial Intelligence
POI: Patient of Interest
DR: Dimensionality reduction
UMAP: Uniform manifold approximation and projection
