# Supplementary material for: Understanding cancer patient cohorts in virtual reality environment for better clinical decisions: a usability study
Source: BMC Med Inform Decis Mak. 2023 Dec 20;23:295. doi: 10.1186/s12911-023-02392-0 (PMC10731816; doi:10.1186/s12911-023-02392-0)
Supplement: Supplementary file 1 — Additional file 1: Supplementary 1. Supplementary tables for the analysis of usability results. [file 12911_2023_2392_MOESM1_ESM.docx]

**Supplementary 1 – Supplementary tables for the analysis of usability results.**

Table 1 Quotes and Responses

| Quotes and Response | Suggestions |
| --- | --- |
| “Genomic visualisation tools took out the complexity of the genomic data and simplified the patterns, which was very useful for the current work.”  “Genomic and cancer data visualisation tools had the advantage of recognising patients’ data in a cohort and finding suitable treatment methods accordingly” | “More information on only one screen instead of jumping among different windows, which makes presenting information among different roles easier”  “More interactions are needed because I want to add new data easily and see more detailed information in a few steps.”  “Artificial Intelligence (AI) is very attractive because the prediction features can assist the decisions”  “We see many applications run on VR devices, and more visualisation features are expected to be added to such applications” |

Table 2 Task Completion Rates

| Participant | Task 1 | Task 2 | Task 3 | Task 4 | Task 5 | Task 6 | Task 7 | Task 8 | Task 9 | Task 10 | Task 11 | Task 12 | Task 13 | Task 14 | Task 15 | Task 16 |
| --- | --- | --- | --- | --- | --- | --- | --- | --- | --- | --- | --- | --- | --- | --- | --- | --- |
| 1 | √ | √ | √ | √ | √ | √ | √ | √ | √ | √ | √ | √ | √ | √ | √ | √ |
| 2 | √ | √ | √ | √ | √ | √ | √ | √ | √ | √ | √ | √ | √ | √ | √ | √ |
| 3 | √ | √ | √ | √ | √ | √ | √ | √ | √ | √ | √ | √ | √ | √ | √ | √ |
| 4 | √ | √ | √ | √ | √ | √ | √ | √ | √ | √ | √ | √ | √ | √ | √ | √ |
| 5 | √ | √ | √ | √ | √ | √ | √ | √ | √ | √ | √ | √ | √ | √ | √ | √ |
| 6 | √ | √ | √ | √ | √ | √ | √ | √ | √ | √ | √ | √ | √ | √ | √ | √ |
| 7 | √ | √ | √ | √ | √ | √ | √ | √ | √ | √ | √ | √ | √ | √ | √ | - |
| 8 | √ | √ | √ | √ | √ | √ | √ | √ | √ | √ | √ | √ | √ | √ | √ | √ |
| 9 | √ | √ | √ | √ | √ | √ | √ | √ | √ | √ | √ | √ | √ | √ | √ | √ |
| 10 | √ | √ | √ | √ | √ | √ | √ | √ | √ | √ | √ | √ | √ | √ | √ | √ |
| 11 | √ | √ | √ | √ | √ | √ | √ | √ | √ | √ | √ | √ | √ | √ | √ | √ |
| 12 | √ | √ | √ | √ | √ | √ | √ | √ | √ | √ | √ | √ | √ | √ | √ | √ |
| 13 | √ | √ | √ | √ | √ | √ | √ | √ | √ | √ | √ | √ | √ | √ | √ | √ |
| 14 | √ | √ | √ | √ | √ | √ | √ | √ | √ | √ | √ | √ | √ | √ | √ | √ |
| 15 | √ | √ | √ | √ | √ | √ | √ | √ | √ | √ | √ | √ | √ | √ | √ | √ |
| 16 | √ | √ | √ | √ | √ | √ | √ | √ | √ | √ | √ | √ | √ | √ | √ | √ |
| 17 | √ | √ | √ | √ | √ | √ | √ | √ | √ | √ | √ | √ | √ | √ | √ | √ |
| 18 | √ | √ | √ | √ | √ | √ | √ | √ | √ | √ | √ | √ | √ | √ | √ | √ |
| 19 | √ | √ | √ | √ | √ | √ | √ | √ | √ | √ | √ | √ | √ | √ | √ | √ |
| 20 | √ | √ | √ | √ | √ | √ | √ | √ | √ | √ | √ | √ | √ | √ | √ | √ |
| Success | 20 | 20 | 20 | 20 | 20 | 20 | 20 | 20 | 20 | 20 | 20 | 20 | 20 | 20 | 20 | 19 |
| Completion Rates | 100% | 100% | 100% | 100% | 100% | 100% | 100% | 100% | 100% | 100% | 100% | 100% | 100% | 100% | 100% | 95% |

Table 3 Mann-Whitney Test: Descriptive Statistics.

| Descriptive Statistics | | | | | | | | |
| --- | --- | --- | --- | --- | --- | --- | --- | --- |
|  | N | Mean | Std. Deviation | Minimum | Maximum | Percentiles | | |
|  |  |  |  |  |  | 25th | 50th (Median) | 75th |
| Overview Interactions | 20 | 2.390 | 1.2814 | 1.3 | 7.0 | 1.500 | 2.000 | 3.000 |
| Analysis | 20 | 14.975 | 4.7806 | 9.5 | 27.5 | 11.000 | 14.500 | 17.500 |
| Exploration | 20 | 7.725 | 3.5780 | 1.5 | 14.5 | 5.000 | 7.250 | 10.875 |
| Two background | 20 | 1.40 | .503 | 1 | 2 | 1.00 | 1.00 | 2.00 |

Table 4 Mann-Whitney Test.

| Ranks | | | | |
| --- | --- | --- | --- | --- |
|  | Two backgrounds | N | Mean Rank | Sum of Ranks |
| Overview Interactions | Medical Domain Users | 12 | 10.38 | 124.50 |
|  | Computing Domain Users | 8 | 10.69 | 85.50 |
|  | Total | 20 |  |  |
| Analysis | Medical Domain Users | 12 | 12.13 | 145.50 |
|  | Computing Domain Users | 8 | 8.06 | 64.50 |
|  | Total | 20 |  |  |
| Exploration | Medical Domain Users | 12 | 7.88 | 94.50 |
|  | Computing Domain Users | 8 | 14.44 | 115.50 |
|  | Total | 20 |  |  |

Table 5 Participants' feedback comments for each question.

| **Question:** | **Feedbacks** | **Strengths** | **Weakness** |
| --- | --- | --- | --- |
| **Is the VR tool useful for distinguishing patient to patient comparisons based on a patient’s genetics? Would you like to give more feedback on the scenarios of the application in your work?** | **Visually compares seemed like a different/better experience**  **It would beautiful way of looking at my own data and feeling how they compare and also germline information**  **The genetic data provided/ reviewed appear to only involved gene expression. This has some relevance to my current work with RNASeq and gene expression at gene fusions. I am also interested in copy number variants and single nucleotides variants and in cancer rNASeq and Chromosome Microarray as diagnose tests.**  **It is useful for 3D visualisation of multidimensional data**  **It is easy to see and interrogate the 3D relationships between patients clustered using various methods.**  **I would need a lot of time to get used to it, but I think it could be very helpful to set an understanding of multi-dimensional data.**  **It was great idea. I can say it is similar to what I am doing in my research and it's great and helpful to present the research outcome in this way so it could be clearer to clinicians.**  **VR tool was useful to me both to get a better view on the data. I also think it is important because it represent a technological avenue for the future as people gain more VR fluency.**  **The location of patients depends on a pool of genes which needs more explanation to be actually useful.**  **Cooling at data in 3D space, hence user can consider lot of dimensions and vectors.** | **A better experience, relevance to daily work, useful for 3D visualisation, easy to see, clearer to clinicians, better view on the data, many dimensions and vectors,** | **Needs to be able to load other data, need time to learn, need more explanation** |
| **Do you feel any physical discomfort at any point while using the software? If so, please describe** | **Headset a little too heavy for me**  **Some discomfort on the eyes.**  **It took a few moments just to familiarise myself with the environment.**  **VR set may be uncomfortable if using for long time**  **I am used to VR and didn't feel sick (one suggestion -- limit speed of Zoom in/out)**  **Slightly discomfort because of the headset because I am not used to it.**  **Surprisingly! (I usually suffer from motion sickness, but I didn't)** | **no motion sickness, didn't feel sick** | **Heavy headset, discomfort on the eyes, need time to familiarise with the environment, Slightly discomfort** |
| **Do you feel eye strain, arm/neck fatigue? If so, please describe:** | **Blurry**  **Eye strain**  **A little neck strain (heavy)**  **grabbing tasks cause fatigue**  **No eye, arm/neck fatigue but did feel disoriented straight after taking off the head piece**  **A little bit of eye strain**  **Didn't feel any fatigue** | **No eye, arm/neck fatigue** | **Blurry, Eye strain, neck strain, disoriented straight** |
| **Do you feel comfortable with how you had to move your hands to navigate within the VR environment? If so, please describe:** | **Gets better with practice. No experience with VR**  **Overall comfortable with moving**  **There was some crossing over of arm movements but again I think getting more familiar with the system would be of benefit.**  **Takes a few minutes to adjust to but then it is fine**  **Everything felt intuitive**  **Certain objects were at very low (ground) level and felt hard to reach with VR headset on. Prefer if visuals are at eye level and higher.** | **Gets better with practice, comfortable with moving, intuitive** | **Some crossing over of arm movements, need time to get used to VR,** |
| **How easy was it to find your way within the virtual environment? Please describe:** | **with time it is fine. Definite learning curve**  **Hard to pick dots**  **take practice**  **will be better with more practice**  **Easy but required reminding of controls**  **Need to adapt and adjust with orientation**  **with some more experience at how to use the system, it would become easier**  **Yes, very good simple to me** | **Definite learning curve, Easy, become easier with practice, simple** | **Hard to pick dots, practice and adaptation required** |
| **Was information such as patient details, labels, legible/clear at all times? Please describe:** | **Text a little hard to read (small)**  **The actual display panels and data visualisation were good and appear relevant to tools.**  **The text on the tools/pads is clear. The text in the small window on the upper left corner is almost unnoticeable, i.e. it is visible, but the brain does not use it and eventually stops registering."**  **Because I use glasses, to different spaces/distances of field was a little bit blur.**  **I needed to wear my glasses as the field of view was fuzzy/blurry. Generally the best view was clear. Moving my head further into the environment improve the quality of the text.**  **"Heatmap was a little blurry sometimes.**  **I had to hold the headset up with one hand to make the text clear** | **good and appear relevant, clear** | **Some small texts, a little bit blur with glasses on, a little blurry, justify headset to make clear** |
| **Do you think the suitable graphical and visual design such as the avatar within the VR environment impacts its ability to find patients for investigation? Please describe:** | **Avatar is good, the circles in selection are good, the cube in marking the patients not seemed very useful**  **I found the size of the console a little too big. The panels that display the data were good**  **It can help to recognize a patient easy**  **Avatars help quickly identify M\|F. But are large relative to other MFO. Would be good if they would quickly add additional info e.g. risk, BMI, age**  **I am red-green color blind, so it was difficult to distinguish some groups.**  **It was easier to determine sex of the patient** | **Good avatar, Good panel design, quickly and easily show gender, help to recognise patients** | **Too big console, not friendly colour blindness, use of cube to mark patients may not be useful, being able to add additional info to avatars potentially useful** |
| **Is the sound helpful in the VR environment?** | **Very helpful**  **Dings are nice, background music helps pass the time**  **To make sure you have done the task like selecting correctly**  **Adds to the VR experience** | **Helpful, nice sound, engaged background music, help to confirm the action, adds to the VR experience** |  |

Table 6 Potentially use in medical domain daily work.

| **Question:** | **Feedbacks** | **Strengths** | **Needs Improvement suggestions** |
| --- | --- | --- | --- |
| **Can you provide any detailed comments of how the VR tool may be used to help researchers or medical doctors make more sense of the complex data comparisons when trying to understand a particular patient?** | **Helpful to get 3D representation of data so get visual cursor to relations, much harder in 2D**  **Demonstrate using different parameter -- Clinical ones (e.g. response to therapy) + biological (genetics) as the training tool --> then try the current version**  **Yes, to improve complex data**  **Aligning physical virtual environments for example some physical surface corresponding to the virtual "surfaces", live tools plate being aligned to the desk/table physical surface brings authenticity (at least feeling connection between environment some sort of continuity of the space).**  **I thought it was helpful feeling emerged/pattern in the data**  **I think it has potential to be used in a practical setting as long as there is a clear clinical Q that can be answered.**  **I think it will be very helpful. Potentially when consulting dimmed features, pathology repeat/data, and complex genomic data. I see great benefit in comparing genomic data with a specific disease cohort to compare the different perspectives and potent outcomes.**  **in cases with complex 3D relationship**  **I'm not a clinician or cancer researcher but feel the ability to integrate is useful. Could be useful to identify interested patient going into VR and the expert interesting patients after wards.**  **Yes, very useful for data comparison, education**  **I think it could be useful to set a more intuitive understanding of similarity between patterns in a complex data space**  **Definitely, it was even so helpful for me (who has some biological background) to compare the patient from different cohorts**  **This application could be useful in multiple fields, where patient to patient, or group is important. I can see applications in clinical and research with Psychology**  **Maybe helpful in searching for genetic disorders, gene expression patterns, risk genes , etc.**  **Current visualisations are good. Potentially adding treatments applied for similar patients can enhance doctor's experience.**  **Definite clinical application possible** | **Helpful, improve complex data, feeling emerged/pattern in the data, potential to be used in a practical setting, useful to identify interested patient, useful for data comparison, useful for data comparison, intuitive understanding, intuitive understanding, useful in multiple fields, useful in multiple fields, useful in multiple fields, great benefit, complex 3D relationship** | **Using different parameter -- Clinical ones (e.g. response to therapy, important to have clear clinical questions to answer** |
