# Supplementary material for: Understanding cancer patient cohorts in virtual reality environment for better clinical decisions: a usability study
Source: BMC Med Inform Decis Mak. 2023 Dec 20;23:295. doi: 10.1186/s12911-023-02392-0 (PMC10731816; doi:10.1186/s12911-023-02392-0)
Supplement: Supplementary file 2 — Additional file 2: Supplementary 2. Questionnaire. [file 12911_2023_2392_MOESM2_ESM.docx]

**Supplementary 2 – Questionnaire**

**Sony Foundation VR Tool Feedback Form**

Participant Number: Interview Date:

**Before Interview**

(a) Can you please give a little background about your experience with evaluating the role of genes and genetic in childhood cancer?

(b) Are you familiar with using Virtual Reality (VR) and its operations? Do you regularly play VR games or have experience in using VR for data analysis? Please describe:

| \| 1. No VR experience \| 2. A little bit VR experience \| 3. Some VR experience \| 4. Lots of VR experience \| 5. Professional VR user \| \| --- \| --- \| --- \| --- \| --- \|   Comments: |
| --- | --- | --- | --- | --- | --- |

-------------------------------------------------------------------------------------------------------

**After Interview:**

1. Is the VR tool useful for distinguishing patient to patient comparisons based on a patient’s genetics? Would you like to give more feedback on the scenarios of the application in your work?

| \| 1. Not Useful at all \| 2. A little bit useful \| 3. Moderately Useful \| 4. Useful \| 5. Very useful \| \| --- \| --- \| --- \| --- \| --- \|   Comments: |
| --- | --- | --- | --- | --- | --- |

1. Do you feel any physical discomfort at any point while using the software? If so, please describe:

| \| 1. Not at all \| 2. A little bit uncomfortable \| 3. Some uncomfortable \| 4. Very uncomfortable \| 5. Very sick \| \| --- \| --- \| --- \| --- \| --- \|   Comments: |
| --- | --- | --- | --- | --- | --- |

1. How long do you think you could use the VR in one sitting? _________
2. Do you feel eye strain, arm/neck fatigue? If so, please describe:

| \| 1. Not at all \| 2. A little bit uncomfortable \| 3. Some uncomfortable \| 4. Very uncomfortable \| 5. Very sick \| \| --- \| --- \| --- \| --- \| --- \|   Comments: |
| --- | --- | --- | --- | --- | --- |

1. Do you feel comfortable with how you had to move your hands to navigate within the VR environment? If so, please describe:

| \| 1. Very uncomfortable \| 2. A little bit uncomfortable \| 3. A little bit comfortable \| 4. Comfortable \| 5. Very comfortable \| \| --- \| --- \| --- \| --- \| --- \|   Comments: |
| --- | --- | --- | --- | --- | --- |

1. How easy was it to find your way within the virtual environment? Please describe:

| \| 1. Not easy at all \| 2. A little bit hard \| 3. Easy but need help \| 4. Easy \| 5. Very Easy \| \| --- \| --- \| --- \| --- \| --- \|   Comments: |
| --- | --- | --- | --- | --- | --- |

1. Was information such as patient details, labels, legible/clear at all times? Please describe:

| \| 1. Not clear at all \| 2. A little bit clear \| 3. Clear but need time \| 4. Legible/Clear \| 5. Very legible/clear \| \| --- \| --- \| --- \| --- \| --- \|   Comments: |
| --- | --- | --- | --- | --- | --- |

1. Do you think the suitable graphical and visual design such as the avatar within the VR environment impacts its ability to find patients for investigation? Please describe:

| \| 1. Not impact at all \| 2. A little bit impact \| 3. Moderately impact \| 4. Impact \| 5. Impact extremely \| \| --- \| --- \| --- \| --- \| --- \|   Comments: |
| --- | --- | --- | --- | --- | --- |

1. Is the sound helpful in the VR environment?

| \| 1. Not helpful \| 2. A little bit helpful \| 3. Moderately helpful \| 4. Helpful \| 5. Very helpful \| \| --- \| --- \| --- \| --- \| --- \|   Comments: |
| --- | --- | --- | --- | --- | --- |

1. Can you provide any detailed comments of how the VR tool may be used to help researchers or medical doctors make more sense of the complex data comparisons when trying to understand a particular patient?

|  |
| --- |

1. Are there any other comments on the VR visualization tool?

|  |
| --- |
